# Lectures on the Physical Phenomena of Living Beings

**Published:** 1848-06

**Authors:** 


Art. IX—*Lectures on the Phenomena of Living Beings.* By Carlo Matteucci, Professor in the University of Pisa. Translated under the superintendence of Jonathan Pereira, M.D. F.R.S. Vice President of the Royal Medical and Chirurgical Society. Philadelphia: Lea & Blanchard. 1848. 12mo., pp. 388.

In the preface to this volume it is stated, that these Lectures were delivered by appointment of the government of Tuscacany, in the University of Pisa, and that in the space of less than four years they have passed through two editions in Italy and one in France. The author, Professor Matteucci, is one of the most eminent of the savans of Italy, his name having been associated with some of the most interesting questions in physiology and chemistry.

The number of lectures embraced in this volume is twenty. The first is introductory, and treats of the general properties and phenomena of living beings. These phenomena are not all explicable by the general properties of matter, and the action of caloric and electricity. Such a conclusion would be as far from the truth as the inference of those who deny, that living beings are at all under the influence of physical agents. There is something about sensation which the laws of physics fail to explain. But because they cannot explain everything we are not to abandon the analogies which the physical sciences offer us. Every day increases the number of vital phenomena which can be referred to chemical action. "The number of catalytic actions in living beings," remarks our author, "is immense."

The second Lecture treats of *molecular attraction, capillary forces, and imbibition.* The laws of capillarity are detailed at length, and imbibition is shown to depend upon the same force. Many of the phenomena of living beings are examples of imbibition.

Endosmose is the subject of the third Lecture—the mutual action of two liquids on each other when separated by a membrane. If we take a glass tube whose lower extremity, closed by a piece of bladder, is expanded into the form of a funnel, and pour into it an aqueous solution of gum or sugar, afterwards immersing the closed extremity in pure water, we find that the water continually passes into the tube by filtration through the membrane. The liquid within becomes elevated, and may even flow over by the upper aperture. At the same time, a certain quantity of the mucilaginous or saccharine liquid escapes through the bladder and mixes with the water. Dutrochet has called the first of these phenomena *endosmose*, and the second *exosmose.*

The principles are applicable to living beings. Thus

"Microscopic observation has now put beyond doubt that, in all tissues, whether vegetable or animal, and in those liquids which are produced by the alteration of organized and living beings, there are constantly found, at a certain epoch, microscopic corpuscles, which have a peculiar characteristic form, and are called elementary or primitive cells. These bodies consist of an exceedingly delicate membrane, which has a spherical form, encloses a liquid, and has on its inner side a small organized body, called the *nucleus* or *cyto-blast.* The cells float at first in a liquid, which Schwann has named *cyto-blastema*, and they ultimately become included in, and almost confounded with it, when this liquid acquires a greater or less density. In different tissues, the elementary cells are more or less closely approximated to each other; the *cyto-blastema*, or intercellular substance, being invariably the bond of union between them. The life of the elementary cells certainly plays the most essential part in the development and preservation of the tissues of living bodies; and, since these cells are found under conditions favorable to endosmose, we have no reason for refusing to admit its existence. A vesicle filled with a liquid, and placed in the midst of another liquid, may act on the outer one, receive the surrounding liquor, and reject the one it had previously contained, by operating in a manner analogous to endosmose."

The action of certain medicines—purgatives for example—seems explicable upon endosmotic principles. Poiseuille found that there was endosmose through animal tissues from the serum of the blood to Seidlitz water, and to solutions of sulphate of soda and common salt. And we know this is precisely what happens when we use these medicines internally; the ejected excrements contain an excess of albumen. In this case endosmose takes place through the capillary vessels of the intestine, from the serum of blood to the saline solution introduced into the alimentary canal. But a still more remarkable fact was discovered by Poiseuille; namely, that the muriate of morphia, when added to a saline solution, very considerably weakens the endosmose from the serum to the solution, and ultimately changes the direction of the current. This corresponds with the constipating effects of opium and its salts, and their action in diarrhœa.

Lecture IV treats of *absorption and exhalation in animals and plants.* It was long a question whether the lymphatic vessels or the veins were endowed with the power of absorption; but Matteucci shows that *all vessels* absorb. Exhalation is effected for the most part, by the same mechanism, and under the same laws as those of absorption. Through the coats of a vessel, capable of imbibing the contained fluid, a portion of it is constantly passing out or exhaling.

"The portion which escapes varies according to the nature of the liquid; that is to say, according to the greater or less facility with which it is imbibed by the tissue of the vessel. In proportion as the sides of this vessel are externally more or less moist, so will the internal liquid flow out with more or less difficulty. The exhalation will be augmented if, in consequence of the very large quantity of contained liquid, the walls of the vessel are subjected to increased pressure. All these particularities of exhalation, which result from what we regarded simply as a physical phenomenon, and which depend on the same principles as absorption, are demonstrated by experimental physiology."

*Digestion* is the subject of the next Lecture, and the author shows that the act is purely physical—a species of *catalytic action* or *action of contact.* Azotized neutral substances, dissolved in the stomach by the acid liquid, or by the catalytic action of pepsine, pass into the blood merely by the imbibition of the coats of the capillary bloodvessels of the stomach. The digestion of these matters is a mere solution effected by an action of contact and an absorption of this solution. Water and alcoholic drinks, introduced into the stomach, are also absorbed.

Amylaceous substances are first converted, by the action of the saliva or the pancreatic juice, into sugar, and then absorbed.

"It is singular that this action requires the presence of a free alkali; for, if the pancreatic juice be acidulated, it ceases to act on starch, but, according to Bernard and Barreswil, acquires the property of acting upon the neutral azotised substances. We must, therefore, conclude, that a single organic substance has the property of dissolving fecula and the azotised neutral matters, provided that we add, when we act on the first, a free alkali, and when on the second, a free acid."

Fatty substances suffer no change in the stomach, but after they have passed out of that viscus are absorbed by the chyliferous vessels. Endosmose is one of the causes which promote this absorption, for it is certain that it could not take place, if the inner sides of the intestines were not bathed with some liquid, for which the fatty bodies had an affinity.

"It is easy to demonstrate, by experiments, that the alkaline condition of the intestinal coats favors this absorption. Fill two funnels with sand, equally shaken down in each. Pour into one, pure water, into the other, an alkaline solution; when the liquids have filtered through, pour an equal quantity of oil on each filter. For several hours, the oil will remain upon the surface of the sand, which has been moistened with pure water; whilst in the other funnel, in which the sand has been moistened with the alkaline solution, the oil will rapidly disappear by imbibition."

The subject of the sixth Lecture is *respiration*, in which, however, we find nothing but what has been given by other physiologists. With all the standard writers of the day, the author holds respiration to be a physico-chemical process—that "the gases dissolved in venous blood are set free by the absorption of other gases; that a portion of the carbonic acid of venous blood is exhaled by the absorption of atmospheric oxygen by this blood; that it is not in the lungs, at least for the most part, that the expired carbonic acid is formed; that this gas exists dissolved in venous blood, and is set free, during the act of respiration, by the presence of oxygen, which is introduced in the same manner as is done by azote or hydrogen in the artificial respiration of these gases; and that, from the experiments of Magnus, it is proved, that the quantity of carbonic acid dissolved in the five pounds (about 6½ lbs. troy) of blood, which traverse the lungs in one minute, is nearly double that which is exhaled in the same time."

The following Lecture treats of *Sanguification, Nutrition, and Animal Heat.* The agency of iron in giving color to blood is investigated, and the result is a doubt whether there is any connexion between the existence of that mineral in the blood and its color. Scherer has recently satisfied himself that he has obtained the coloring matter of the blood entirely free from iron. The following suggestion respecting the action of the blood globules is ingenious.

"The blood globules, making no part of the tissue, though being essential to nutrition, may, with a certain amount of probability, be regarded as the *catalytic* body which promotes the transformation and continual renovation of the tissues. An analogy of this characteristic of the globules is to be found in the necessity which they experience of being charged with oxygen in order to acquire this property."

During the circulation of the blood a continual combustion is going on, in which both carbon and hydrogen, and phosphorus, also, according to Rees, undergo oxidation. Carbonic acid, urea, water and other compounds, result, and heat is necessarily evolved. This is the source of animal heat, according to M. Matteucci.

We pass over the next chapter on the *Phosphorescence* of organized beings, simply remarking that it contains much curious matter on that obscure subject.

The ninth Lecture is devoted to the consideration of the *Electrical current of Muscles.* Not only are heat and light evolved in the living organisms by chemical actions, but electricity also. The following easily executed experiment proves the existence of an electrical current when different parts of the same muscular mass are made to connect by means of a conducting body.

"A frog is prepared according to the usual method of Galvani; that is, we cut it through the middle of its pelvis, separate carefully all the muscles of the thigh, and divide one of the lumbar plexuses as it passes out of the vertebral column. We then have the leg of a frog united to its long nervous filament, composed of the lumbar plexus, and of its prolongation in the thigh, that is to say, of the crural nerve. The frog, thus prepared, and which I have called the *galvanoscopic frog*, is very useful in researches on the electric current. For this purpose we introduce the claw of a frog into a glass tube covered with an insulating varnish, take hold of the tube with the hand, and afterwards bring any two parts of the body, whose electric state we wish to examine, in contact with two different and sufficiently distant points of the nervous filament of the galvanoscopic frog.

"If we take the precaution of not touching the body with any portion of the muscle of the leg, and if the limb be well insulated from the hand, we may be certain that the contraction which the galvanoscopic frog suffers, is due to a current produced in the body touched, and that the nerve only conducts it, and renders it evident by the contraction of the muscle."

The existence of this current is further shown by the following experiment:

"Furnished with a frog, thus prepared, I take a living animal, a pigeon for example, slightly cut its pectoral muscle, after having carefully removed the integuments, and introduce into the wound the nerve of the galvanoscopic frog.

"You observe the contraction of the frog. If you reflect on the arrangement, you will be satisfied that it is absolutely necessary to touch two distinct parts of the pectoral muscle of the pigeon, with two different parts of the nervous filament. If I apply the extremity of the nerve to the bottom of the wound, and another portion of the nerve to the lips of the wound, or, better still, to the external surface of the muscle, the frog continually contracts. This experiment clearly demonstrates the presence of an electric current, which circulates in the nerve, since it is necessary to form a circuit in which the nerve forms a part. If you have any doubt that the contractions of the frog are really excited by a current due to the different parts of the muscle of the animal, you will soon be convinced, by finding that no contractions are produced when I touch two different parts of the nerve with one liquid, or with a perfectly homogenous conducting body."

What are the sources of this muscular current? Matteucci does not believe it is explicable upon Liebig's hypothesis—the combination of the free acid of the muscles with the alkili of the lymph and blood. His explanation of the phenomenon is as follows:

"The origin of this current resides in the electric conditions which are produced by the chemical actions of the nutrition of the muscle. The blood charged with oxygen, and the muscular fibre, which becomes transformed on contact with this liquid, compose the elements of a pile: they are the liquid acid and zinc. In the normal condition of the muscle, there can only be molecular currents produced by the formation and destruction of opposite electrical conditions in the same points; but if a great number of points of the muscular fibre be put, by means of a good conductor, in communication with others of a different nature, which do not suffer the same chemical action on the part of the blood, the electric current should then circulate. It is this fact, furnished to us by experiment, which proves at the same time the development of electricity in the living muscle, and the impossibility for the electric current to circulate in the masses of this muscle in the natural condition."

We are here compelled to take leave for the present of these instructive lectures.

